# Simultaneous Removal of Cr(VI) and Phenol from Water Using Silica-di-Block Polymer Hybrids: Adsorption Kinetics and Thermodynamics

**DOI:** 10.3390/polym14142894

**Published:** 2022-07-16

**Authors:** Jia Qu, Qiang Yang, Wei Gong, Meilan Li, Baoyue Cao

**Affiliations:** Shaanxi Key Laboratory of Comprehensive Utilization of Tailings Resources, Shaanxi Engineering Research Center for Mineral Resources Clean & Efficient Conversion and New Materials, Shangluo University, Shangluo 726000, China; yq_sust@163.com (Q.Y.); gongwei-com@escience.cn (W.G.); liecho2009@163.com (M.L.); cby0406@163.com (B.C.)

**Keywords:** silica, amphipathic, block copolymer, adsorption, Cr(VI), phenol

## Abstract

Heavy metal ions and organic pollutants often coexist in industrial effluents. In this work, silica-di-block polymer hybrids (SiO_2_-g-PBA-b-PDMAEMA) with two ratios (SiO_2_/BA/DMAEMA = 1/50/250 and 1/60/240) were designed and prepared for the simultaneous removal of Cr(VI) and phenol via a surface-initiated atom-transfer radical polymerization process using butyl methacrylate (BA) as a hydrophobic monomer and 2-(Dimethylamino)ethylmethacrylate (DMAEMA) as a hydrophilic monomer. The removal efficiency of Cr(VI) and phenol by the hybrids reached 88.25% and 88.17%, respectively. The sample with a larger proportion of hydrophilic PDMAEMA showed better adsorption of Cr(VI), and the sample with a larger proportion of hydrophobic PBA showed better adsorption of phenol. In binary systems, the presence of Cr(VI) inhibited the adsorption of phenol, yet the presence of phenol had a negligible effect on the adsorption of Cr(VI). Kinetics studies showed that the adsorption of Cr(VI) and phenol fitted the pseudo-second-order model well. Thermodynamic studies showed that the adsorption behavior of Cr(VI) and phenol were better described by the Langmuir adsorption isotherm equation, and the adsorption of Cr(VI) and phenol were all spontaneous adsorptions driven by enthalpy. The adsorbent still possessed good adsorption capacity for Cr(VI) and phenol after six adsorption–desorption cycles. These findings show that SiO_2_-g-PBA-b-PDMAEMA hybrids represent a satisfying adsorption material for the simultaneous removal of heavy metal ions and organic pollutants.

## 1. Introduction

Water pollution is a worldwide problem that requires urgent attention and prevention. Heavy metal ions [1,2] and organic pollutants [3,4] are common pollutants in water which pose a serious threat to human health and the ecological environment. The most commonly toxic heavy metal ions in water include Cr(VI) [5,6,7], As(III) [8], Cd(II) [9], Hg(II) [10], Pb(II) [11], Cu(II) [12], Zn(II) [13], etc. The main organic pollutants include phenolic compounds [14,15], benzene compounds [16,17], halohydrocarbons [18,19] and so on. Among various water purification and recycling technologies, adsorption is a fast, effective, easy to operate, inexpensive, and a universal method [20,21]. Current research on adsorbent development mainly focuses on the adsorption of a certain type of pollutant like heavy metal ions or organic pollutants. But in practical application, adsorption materials are required to have good adsorption performance for different types of pollutants [22,23]. Therefore, it is urgent to design a new type of adsorption material which can adsorb metal ions and organic molecules simultaneously and efficiently. 

Organic/inorganic nano-hybrids have significant advantages in terms of having inorganic nanoparticles with high specific surface area, high mechanical strength, high thermal stability, and durability [24,25]. What’s more, by means of molecular design, the organic polymer chains of organic/inorganic nano-hybrids can adsorb different kinds of pollutants at the same time via van der Waals forces [26], hydrogen bonding [27], charge interactions [28], complexation [29], and other driving forces [30,31]. Therefore, organic/inorganic nano-hybrids are deemed to be an ideal adsorption material [32,33,34].

Except for the usual advantages of inorganic nanoparticles, nano-silica is low-cost and easily functionalized [35,36,37]. On the other hand, block co-polymerization is a useful method for getting functional co-polymers since block co-polymer has the superiorities of a clear chemical structure and narrow molecular weight distribution [38,39,40]. In this work, a new kind of silica-di-block polymer hybrid adsorbent, SiO_2_-g-PBA-b-PDMAEMA, was prepared by surface-initiated atom-transfer radical polymerization (SI-ATRP) [41] using butyl methacrylate (BA) as a hydrophobic monomer and 2-(Dimethylamino)ethylmethacrylate (DMAEMA) as a hydrophilic monomer. Interaction between the hydrophobic block PBA and the organic pollutants was expected, and the hydrophilic functional segment PDMAEMA was introduced into the adsorbent for its affinity with heavy metal ions. The structure of SiO_2_-g-PBA-b-PDMAEMA was characterized by FTIR, GPC, and TEM. The adsorption kinetics and thermodynamics of Cr(VI) and phenol in water were studied, and the competitive-adsorption behavior of binary systems of Cr(VI) and phenol was discussed. 

## 2. Materials and Methods

### 2.1. Materials

Nano-silica (SiO_2_, > 99%wt, a mean particle diameter of 20 nm and a specific surface area of 120 m^2^·g^−1^) was purchased from Hai Tai Nano (Nanjing, China) and used as received. 2-(Dimethylamino)ethylmethacrylate (DMAEMA, 99%) was purchased from Aladdin (Shanghai, China), which was dried over calcium hydride (CaH_2_, 95%, Aladdin) for 24 h and distilled under reduced pressure before use. Butyl acrylate (BA, 99%, Aladdin) was rinsed with 5 wt% NaOH (97%, Aladdin) aqueous solution and dried over CaH_2_ for 24 h. Tetrahydrofuran (THF, 99%, Aladdin) and cyclohexanone (CYC, 99.5%, Aladdin) were stirred over CaH_2_ for 24 h at room temperature and distilled under reduced pressure prior to use. Triethylamine (TEA, 99%, Aladdin) was purified by distillation after drying over CaH_2_. Cuprous chloride (CuCl, 97%, Aladdin) was purified before use [42]. (3-aminopropyl) triethoxysilane (APTES, 99%), 2-bromoisobutyrylbromide (BiBB, 98%), N,N,N′,N′,N″-pentamethyldiethylenetriamine (PMDETA, 99%), copper chloride (CuCl_2_, 98%), phenol (C_6_H_6_O, 99%), potassium dichromate (K_2_Cr_2_O_7_, 98%), hydrofluoric acid (HF, 40%), 1,5-diphenyl carbazide (98%) and ethanol (99.5%) were supplied by Aladdin and used as-received without further purification. Nitric acid (HNO_3_, 68%) was supplied by Foshan Huaxisheng Chemical Co. Ltd. (Foshan, China) and used as-received without further purification.

### 2.2. Preparation of Silica Initiator SiO_2_-Br

**Scheme 1:**
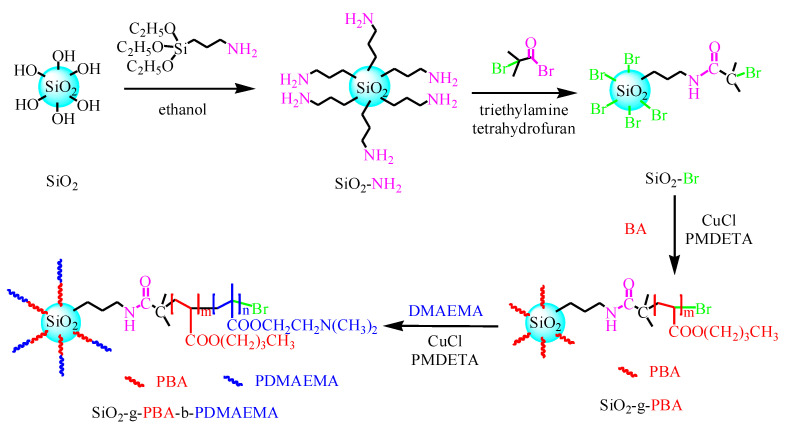
Synthesis of SiO_2_-g-PBA-b-PDMAEMA.

The first step was to prepare the amino-modified nano-silica (SiO_2_-NH_2_). An amount of 2.7 g SiO_2_ was dispersed in a mixture of 87 mL water and 63 mL ethanol for 30 min. Then, 10.5 mL APTES was dissolved in 24 mL ethanol and then added to the above suspension. The pH of the system was adjusted to 10 by ammonium hydroxide, and the reaction was kept for 24 h at 50 °C. After centrifugation, the lower solid layer was alternately washed and centrifuged with water and ethanol. SiO_2_-NH_2_ was obtained after vacuum-drying at 50 °C for 24 h with a yield of 78%. The second step was to bring the bromine atoms into SiO_2_-NH_2_. An amount of 0.5 g SiO_2_-NH_2_ and 10 mL THF was added into a dried Schlenk flask and dispersed by ultrasound for 30 min. Under the condition of an ice bath, the Schlenk flask was filled with N_2_ via three vacuum/N_2_ cycles and the suspension was stirred for 30 min. An amount of 1.5 mL TEA was added subsequently and then mixed with the solution of 3 mL BiBB and 40 mL THF was injected dropwise into the flask to react for 4 h in an ice bath followed by 48 h of reaction in a water bath at 35 °C. After centrifugation, the lower solid layer was alternately washed and centrifuged with water and ethanol. The silica surface-initiator (SiO_2_-Br) was obtained after vacuum-drying at 50 °C for 36 h with a yield of 72%. The synthesis scheme of the silica initiator SiO_2_-Br is given in Scheme 1.

### 2.3. Preparation of SiO_2_-g-PBA-b-PDMAEMA Hybrids by SI-ATRP

SiO_2_-g-PBA-b-PDMAEMA hybrids were obtained via a one-step ATRP. CuCl and CuCl_2_ were added into a dried Schlenk flask, and the flask was sealed with a rubber septum prior to three vacuum/N_2_ cycles. BA and PMDETA were injected into the flask, followed by the suspension of SiO_2_-Br dispersed in cyclohexanone; the reaction was performed at 90 °C for 24 h. After cooling to 70 °C, the cyclohexanone solution of DMAEMA was injected, and the reaction was performed at 70 °C for 24 h. SiO_2_-g-PBA-b-PDMAEMA was finally obtained by centrifugation and washing with THF. The synthesis scheme of SiO_2_-g-PBA-b-PDMAEMA is also given in Scheme 1, and the detailed recipes of polymerization are listed in Table 1.

### 2.4. Characterization of Initiator and Hybrids

The chemical structures for SiO_2_-Br and SiO_2_-g-PBA-b-PDMAEMA were characterized by Fourier transform infrared spectroscopy (FTIR, Nicolet-380, Thermo Electron Corporation, USA) in a spectral range of 4000–400 cm^−1^. The molecular weights of PBA-b-PDMAEMA cleaved from SiO_2_-g-PBA-b-PDMAEMA by hydrofluoric acid were measured by gel permeation chromatograph (GPC, PL-GPC50, Agilent, USA), equipped with the PL gel-MIXEDC chromatographic column and were calibrated by polystyrene standards. THF was used as the eluent, and the flow rate was 0.5 mL·min^−1^. The particles of SiO_2_-g-PBA-b-PDMAEMA were observed by transmission electron microscopy (TEM, Talos F200X, FEI, Czech Republic) at an acceleration voltage of 200 kV. Samples were ultrasounded in water for 30 min before measurement. The surface-grafted density of SiO_2_-Br was obtained by thermogravimetric analyzer (TGA, STA449, NETZSCH, Germany) under N_2_ atmosphere using a heating rate of 10 °C·min^−1^ from 25 °C to 1000 °C.

### 2.5. Batch Adsorption Study 

The batch mode adsorption studies were carried out by adding 0.05 g SiO_2_-g-PBA-b-PDMAEMA into 50 mL Cr(VI) aqueous solutions (prepared by dissolving K_2_Cr_2_O_7_ in deionized water and mostly existing as HCrO_4_^−^ and CrO_4_^2^^−^ [43]) or 50 mL phenol aqueous solutions on a shaker at 120 rpm under controlled pH of 6. After thermostatic shaking for a certain period of time, supernatant obtained by centrifugation was analyzed to detect the concentration of Cr(VI) or phenol residues. 

The concentration of residue Cr(VI) was determined using a ultraviolet-visible spectrophotometer (UV-Vis, Cary 5000, Agilent, USA) and developing a purple-violet color with 1,5-diphenyl carbazide in acidic solution as a complexing agent [44,45]. The absorbance of the purple-violet colored solution was read at 540 nm after 10 min of color development.

The concentration of phenol residue was determined using a UV-Vis spectrophotometer with maximum absorption wavelength of 270 nm [46,47].

The equilibrium adsorption amount *Q*_e_ (mg·g^−1^) of SiO_2_-g-PBA-b-PDMAEMA of Cr(VI) or phenol was calculated according to Equation (1) [48]. The percentage removal *η*(%) was calculated according to Equation (2) [4]:

*Q* = (*C*_0_ − *C*_t_)*V*/*W* (1)

*η*= 100(*C*_0_ − *C*_t_)/*C*_0_ (2)

where *C*_0_ (mg·L^−1^) and *C*_t_ (mg·L^−1^) are the initial concentrations and equilibrium concentrations of Cr(VI) or phenol, respectively, and *V* (L) is the volume of the solution and *W* (g) is the weight of the adsorbent.

### 2.6. Adsorption Kinetics

Adsorption kinetics were investigated to evaluate both the rate of Cr(VI) or phenol adsorption and the equilibrium time required for the adsorption isotherm. Experiments were conducted under an initial Cr(VI) or phenol concentration of 100 mg·L^−1^, a controlled temperature of 298 K, and a controlled time *t* (5 min, 10 min, 20 min, 30 min, 60 min, 90 min, 120 min, 180 min, and 240 min). 

Adsorption rate was analyzed using two kinetic models, i.e., the pseudo-first-order model and pseudo-second-order model. The pseudo-first-order kinetic model is expressed by Equation (3) [48]:

ln(*Q*_e_ − *Q*_t_) = −*k*_1_*t* + ln*Q*_e_ (3)

where *Q*_e_ (mg·g^−1^) and *Q*_t_ (mg·g^−1^) are the amount of Cr(VI) or phenol adsorbed at the equilibrium and time *t* (min), respectively, and *k*_1_ (min^−1^) is the pseudo-first-order rate constant for the adsorption process. Values of *k*_1_ were calculated from the slope of the ln(*Q*_e_−*Q*_t_) vs. *t* plot. Values of *Q*_e_ were calculated from the intercept of the ln(*Q*_e_−*Q*_t_) vs. *t* plot.

The pseudo-second-order kinetic model is expressed by Equation (4) [48].

*t*/*Q*_t_ = *t*/*Q*_e_ + 1/(*k*_2_*Q*_e_^2^) (4)

where *Q*_e_ (mg·g^−1^) and *Q*_t_ (mg·g^−1^) are the amount of Cr(VI) or phenol adsorbed at the equilibrium and time *t* (min), respectively, and *k*_2_ (g·mg^−1^·min^−1^) is the pseudo-second-order rate constant for the adsorption process. Values of *k*_2_ and *Q*_e_ were calculated from the slope and intercept of the *t*/*Q*_t_ vs. *t* plot.

### 2.7. Adsorption Isotherms

The adsorption isotherms were measured by controlling Cr(VI) or phenol concentration at 10 mg·L^−1^, 20 mg·L^−1^, 50 mg·L^−1^, 100 mg·L^−1^, 150 mg·L^−1^, 200 mg·L^−1^, and 300 mg·L^−1^, respectively. After 120 min of equilibrium, the supernatant was analyzed for the concentration of residual Cr(VI) or phenol.

Two isotherm models were investigated, i.e., the Langmuir model and the Freundlich model. The Langmuir model is expressed by Equation (5) [48]:

*C*_e_/*Q*_e_ = 1/(*Q*_m_*K*_L_) + *C*_e_/*Q*_m_ (5)

where *Q*_e_ is the equilibrium adsorption capacity (mg·g^−1^), *C*_e_ is the equilibrium concentration in the solution (mg·L^−1^), *Q*_m_ is the maximum adsorption capacity (mg·g^−1^), and *K*_L_ is the Langmuir adsorption isotherm constant (L·mg^−1^). Values of *Q*_m_ were calculated from the slope of the *C*_e_/*Q*_e_ vs. *C*_e_ plot. Values of *K*_L_ were calculated from the intercept of the *C*_e_/*Q*_e_ vs. *C*_e_ plot.

The Freundlich model is expressed by Equation (6) [48]:

ln*Q*_e_ = ln*K*_f_ + (1/*n*)ln*C*_e_ (6)

where *Q*_e_ is the equilibrium adsorption capacity (mg·g^−1^) and *C*_e_ is the equilibrium concentration in the solution (mg·L^−1^). *K*_f_ is the Freundlich adsorption isotherm constant [(mg·g^−1^) (L·mg^−1^)^1/n^)]. The term 1/*n* is related to the magnitude of the adsorption driving force. Values of *n* were calculated from the slope of the ln*Q*_e_ vs. ln*C*_e_ plot. Values of *K*_f_ were calculated from the intercept of the ln*Q*_e_ vs. ln*C*_e_ plot.

### 2.8. Thermodynamic Study

Three basic thermodynamic parameters were studied: the Gibbs free energy of adsorption (Δ*G*, J·mol^−^^1^), the enthalpy change (Δ*H*, J·mol^−^^1^), and the entropy change (Δ*S*, J·mol^−^^1^·K^−^^1^). 

Δ*G* was calculated according to Equation (7) [48,49,50]:

Δ*G* = −*RT*ln*K* (7)

where *R* is the universal gas constant (8.314 J·mol^−1^·K^−^^1^), *T* is the absolute temperature (K), and *K* is the derived Langmuir equilibrium constant.

Δ*H* and Δ*S* were calculated according to the van’t Hoff equation, which is expressed by Equation (8) [48]:

ln*K* = −Δ*H*/(*RT*) + Δ*S*/*R* (8)

where *K* is the derived Langmuir equilibrium constant, *R* is the gas constant (8.314 J·mol^−^^1^·K^−^^1^), and *T* is the absolute temperature (K).

According to Equation (8), a linear relationship exists between ln*K* and 1/*T*. Δ*H* and Δ*S* were calculated from the slope and the intercept of the ln*K* vs. 1/*T* plot, respectively.

### 2.9. Recovery Experiments

Six adsorption-desorption cycles were carried out to evaluate the recovery performance of the adsorbent. The adsorption of Cr(VI) or phenol was conducted at an initial concentration of 100 mg·L^−^^1^, a controlled pH of 6, and a controlled temperature of 298 K. After adsorption equilibrium, the adsorbed Cr(VI) was removed from the adsorbent using 0.1 mol·L^−^^1^ nitric acid. The adsorbent was washed with deionized water and vacuum-dried before use. The adsorbed phenol was removed from the adsorbent using ethanol. The adsorbent was washed with deionized water and vacuum-dried before use.

### 2.10. Binary Systems Competitive Adsorption

The binary-system adsorption studies were carried out using batch mode adsorption to understand the competitive behavior between Cr(VI) and phenol onto SiO_2_-g-PBA-b-PDMAEMA. The adsorption experiments were conducted by controlling the initial Cr(VI) concentration of 100 mg·L^−^^1^ and varying the initial phenol concentration stepwise or by controlling the initial phenol concentration of 100 mg·L^−^^1^ and varying the initial Cr(VI) concentration stepwise. The concentration of Cr(VI) residue was measured according to the diphenyl carbazide spectrophotometric method using a UV-Vis spectrophotometer at a wavelength of 540 nm. The concentration of phenol residue was determined using a UV-Vis spectrophotometer at a wavelength of 270 nm. The equilibrium adsorption amount *Q*_e_ (mg·g^−1^) was calculated according to Equation (1).

## 3. Results and Discussion

### 3.1. Adsorbent Characterizations

**Figure 1:**
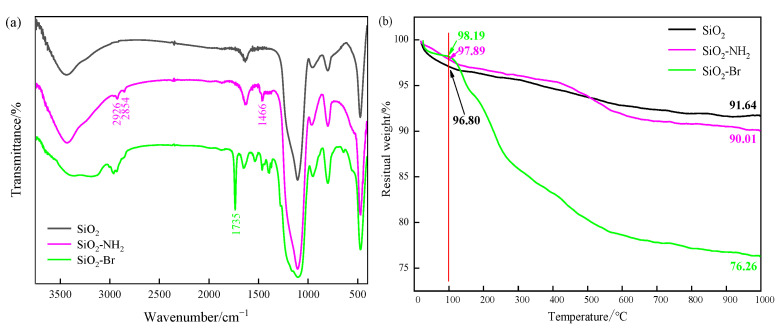
(**a**) IR spectra and (**b**) TGA curves of SiO_2_, SiO_2_-NH_2_, and SiO_2_-Br.

The preparation of the silica initiator SiO_2_-Br was confirmed by FTIR and TGA analysis shown in Figure 1. The new peaks in the FTIR spectrum of SiO_2_-NH_2_ at 2926 cm^−1^, 2854 cm^−1^, and 1466 cm^−1^ were attributed to the C-H vibration of APTES, which proved the grafting of APTES to SiO_2_. Compared with the FTIR spectrum of SiO_2_-NH_2_, the new peak in the spectrum of SiO_2_-Br at 1735 cm^−1^ was attributed to the C=O vibration of BiBB, which proved the grafting of BiBB onto SiO_2_-NH_2_. Furthermore, the TGA curves showed that the weight loss from 100 °C to 1000 °C for SiO_2_, SiO_2_-NH_2_, and SiO_2_-Br was 5.16%, 7.88%, and 21.93%, respectively; the weight loss below 100 °C was owed to the removal of the water absorbed physically. According to these results, the graft density was calculated at 0.81 mmol·g^−1^ for SiO_2_-Br by the content of Br in SiO_2_-Br.

**Figure 2:**
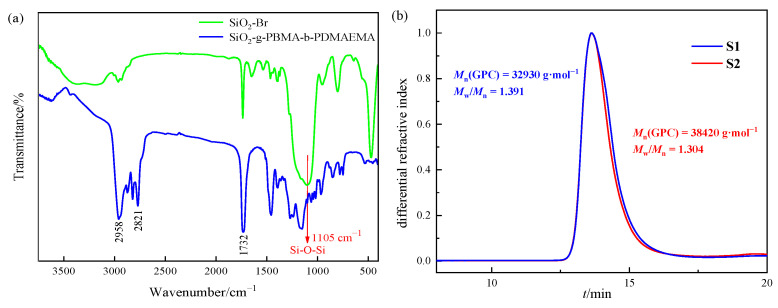
(**a**) IR spectra and (**b**) GPC curves of SiO_2_-g-PBA-b-PDMAEMA.

The verification of BA and DMAEMA grafting onto the silica surface was obtained by the FTIR and GPC analysis of SiO_2_-g-PBA-b-PDMAEMA shown in Figure 2. The peaks in the FTIR spectrum at 2958 cm^−1^ and 2821 cm^−1^ were attributed to the stretching vibration of C-H in the PBA and PDMAEMA chains. The peaks at 1732 cm^−1^ were attributed to the stretching vibration of C=O. The peaks at 1105 cm^−1^ were attributed to the stretching vibration of Si-O-Si. The FTIR spectrum of SiO_2_-g-PBA-b-PDMAEMA showed that PBA and PDMAEMA chains were successfully grafted onto the silica initiator SiO_2_-Br. Furthermore, after etching by hydrofluoric acid, the molecular weights (*M*_n_) of PBA-b-PDMAEMA cleaved from SiO_2_-g-PBA-b-PDMAEMA were 32,930 g·mol^−1^ for S1 and 38,420 g·mol^−1^ for S2, which were close to the theoretical value for the single arm (45711 g·mol^−1^ for S1 and 45,421 g·mol^−1^ for S2, calculated from Table 1). The polydispersity indexes (PDIs) were 1.304 for S1 and 1.391 for S2, which revealed the narrow distribution of the molecular weights. GPC results illustrated that the polymerizations initiated by SiO_2_-Br were all typically controllable ATRP, and the PBA-b-PDMAEMA chains were grafted as expected.

**Figure 3:**
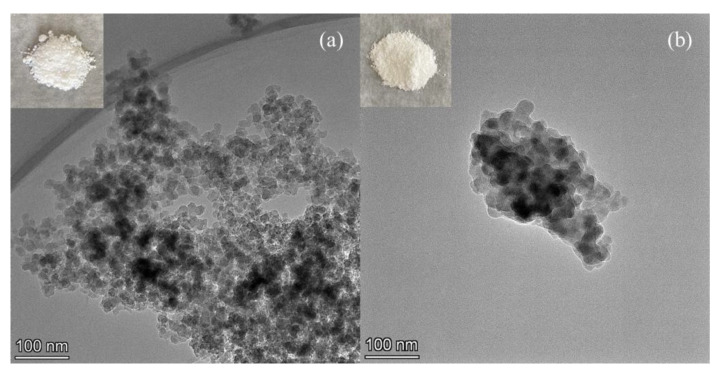
TEM images of (**a**) SiO_2_ and (**b**) SiO_2_-g-PBA-b-PDMAEMA.

TEM images of SiO_2_ and SiO_2_-g-PBA-b-PDMAEMA at 100 nm are shown in Figure 3. The particle size of SiO_2_ was about 20 nm. After grafting via PBA-b-PDMAEMA chains, the silica-di-block polymer hybrids formed spherical particles with a significantly increased diameter of 35–40 nm. Since the PBA-b-PDMAEMA chains stretched in water to some extent, the contact between the pollutants and the adsorbent in water could be improved.

### 3.2. Single Systems Adsorption Kinetics

**Figure 4:**
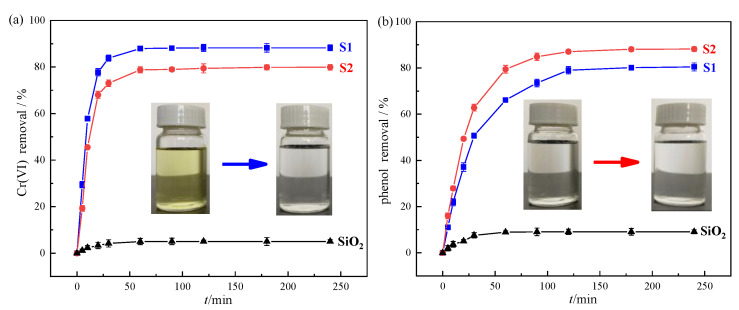
Adsorption kinetics of (**a**) Cr(VI) or (**b**) phenol on SiO_2_-g-PBA-b-PDMAEMA and SiO_2_ (C_0_ = 100 mg·L^−1^, pH = 6, T = 298 K, adsorbent concentration = 1 g·L^−1^).

Single system adsorption kinetics curves are shown in Figure 4. As a comparison, adsorption using bare nano-silica was also discussed. It could be seen from Figure 4a that Cr(VI) was adsorbed by SiO_2_-g-PBA-b-PDMAEMA rapidly within 0–20 min, and the removal efficiency of Cr(VI) tended to be a constant after 60 min. The removal efficiency vs. *t* plot suggested to us that it took about 60 min for Cr(VI) to reach adsorption equilibrium on the surface of the adsorbent. The removal efficiency of Cr(VI) on S1 was 88.25%, which was more than 17-times higher than the removal efficiency using bare nano-silica (5.01%). In addition, the removal efficiency of Cr(VI) on S1 was higher than that of Cr(VI) on S2 (79.88%). This was probably because of the larger proportion of PDMAEMA in S1, which might have a stronger affinity for water-soluble ions. Figure 4b shows that phenol was adsorbed by SiO_2_-g-PBA-b-PDMAEMA rapidly within 0–30 min, and the removal efficiency of phenol tended to be constant after 120 min. This suggested to us that the adsorption equilibrium was achieved within 120 min for phenol on the adsorbent. The removal efficiency of phenol on S2 was 88.17%, which was more than 9-times higher than the removal efficiency using bare nano-silica (9.09%) and was higher than the removal efficiency of phenol on S1 (80.44%). The adsorption capacity of SiO_2_-g-PBA-b-PDMAEMA for phenol greatly improved after functionalization, and the larger proportion of hydrophobic PBA in S2 might be more conducive to the absorption of organic pollutants.

**Figure 5:**
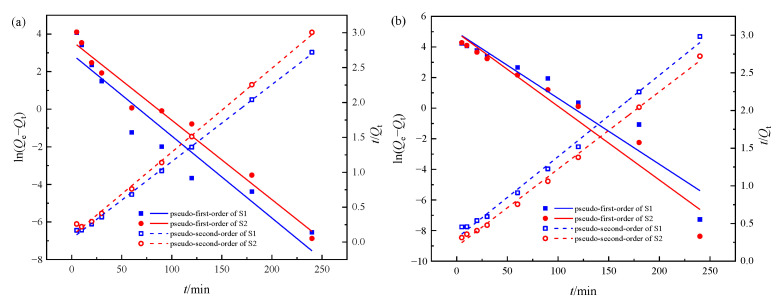
Fitting curves of adsorption kinetics for (**a**) Cr(VI) and (**b**) phenol on SiO_2_-g-PBA-b-PDMAEMA.

The adsorption kinetics data obtained experimentally were fitted to the pseudo-first-order and pseudo-second-order models. The fitting results are shown in Figure 5 and Table 2.

The pseudo-second-order model showed much better fitting to Cr(VI), and phenol adsorption data with higher correlation coefficients (*R*^2^ > 0.99) and a better agreement between experimental (*Q*_e,exp_) and calculated (*Q*_e,cal_) values is also exhibited in Table 2. This indicated that the adsorption rates of Cr(VI) and phenol onto SiO_2_-g-PBA-b-PDMAEMA were controlled by chemical processes [9]. The rate constant *k*_2_ of Cr(VI) on S1 (0.00242 g·mg^−1^·min^−1^) was relatively higher than that of Cr(VI) on S2 (0.00167 g·mg^−1^·min^−1^), indicating a faster uptake of Cr(VI) onto S1. Similarly, the rate constant *k*_2_ of phenol on S2 (0.00054 g·mg^−1^·min^−1^) was relatively higher than that of phenol on S1 (0.00038 g·mg^−1^·min^−1^), indicating a faster uptake of phenol onto S2.

### 3.3. Single Systems Adsorption Isotherms and Thermodynamic Study

**Figure 6:**
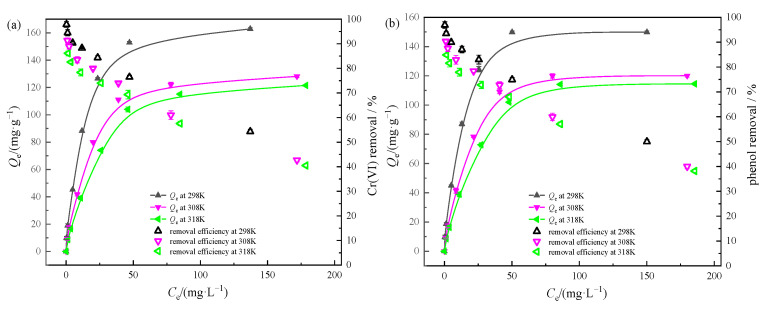
Adsorption isotherms of (**a**) Cr(VI) on S1 and (**b**) phenol on S2.

The adsorption isotherms of Cr(VI) or phenol in aqueous solution by SiO_2_-g-PBA-b-PDMAEMA at different temperatures are shown in Figure 6. The equilibrium adsorption capacity of Cr(VI) or phenol rose with the increase of Cr(VI) or phenol equilibrium concentration and then tended to be constant, i.e., the saturated adsorption capacity. On the other hand, the equilibrium adsorption capacity of Cr(VI) or phenol decreased with the increase in adsorption temperature, indicating that the adsorption of Cr(VI) and phenol were all exothermic processes under experimental conditions. Decreasing the temperature was favorable for the adsorption of Cr(VI) or phenol on SiO_2_-g-PBA-b-PDMAEMA. 

**Figure 7:**
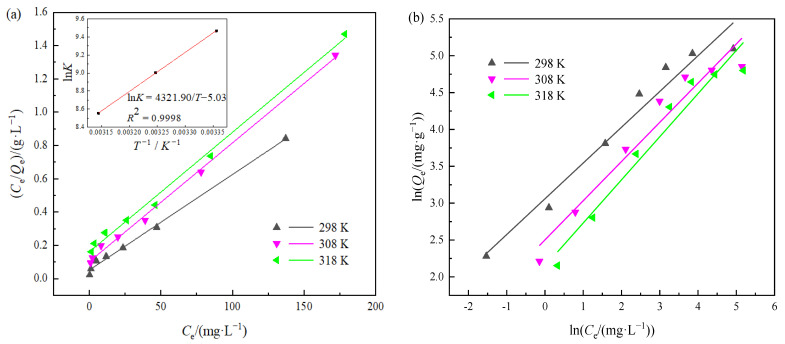
(**a**) Langmuir and (**b**) Freundlich adsorption isotherms for the adsorption of Cr(VI) on S1.

Fitting curves and adsorption isotherm constants for Cr(VI) adsorption on S1 are shown in Figure 7 and Table 3. According to the desired *R*^2^ greater than 0.99, the adsorption behavior of Cr(VI) on the adsorbent surface is deemed to be better described by the Langmuir adsorption isotherm equation, indicating that Cr(VI) was mainly adsorbed by monolayer. This was because, after the monolayer adsorption of Cr(VI) on the surface of the adsorbent, the excess Cr(VI) did not easily approach the surface of the adsorbent due to the existence of electrostatic repulsion. According to the fitting parameters in Table 3, the saturated adsorption capacity of Cr(VI) at 298 K reached 174.22 mg·g^−1^. The *n* values fitted by the Freundlich adsorption isotherm equation were all greater than 1, indicating that the adsorption of Cr(VI) on S1 were beneficial adsorption processes [51]. At 298 K, the *n* value was between 2 and 10, indicating that the adsorption of Cr(VI) on S1 more easily occurred at room temperature [52]. 

According to the Langmuir adsorption isotherm constant *K*_L_, the thermodynamic equilibrium constant *K* was obtained by getting rid of the unit. The plot of ln *K* vs. 1/*T* for thermodynamic parameter calculation is shown in the inset picture of Figure 7a. The thermodynamic parameters calculated for Cr(VI) adsorption on S1 are shown in Table 3. Δ*H* < 0 indicated that the adsorption was an exothermic process. Reducing the temperature was conducive to adsorption, and the adsorption was enthalpy-driven adsorption-type behavior. Δ*S* < 0 indicated that the adsorption of Cr(VI) on S1 reduced the disorder degree of the system, and the adsorption was not entropy-driven adsorption-type behavior. Δ*G* ranged from −23.41 kJ·mol^−1^ to −22.58 kJ·mol^−1^. The negative values of Δ*G* indicated that the adsorption was a spontaneous process. Moreover, it could be inferred that the adsorption driving force was more than the typical physical interactions since studies have shown that the Δ*G* of physical adsorption was −20–0 kJ·mol^−1^ and the Δ*G* of chemical adsorption was −400–−80 kJ·mol^−1^ [53]. Δ*G* of between −40 kJ·mol^−1^ and −20 kJ·mol^−1^ suggested to us that the main adsorption force might have been the electrostatic coulombic attraction [54]; this was consistent with our previous work on the adsorption of Cr(VI) to PDMAEMA chains [28].

**Figure 8:**
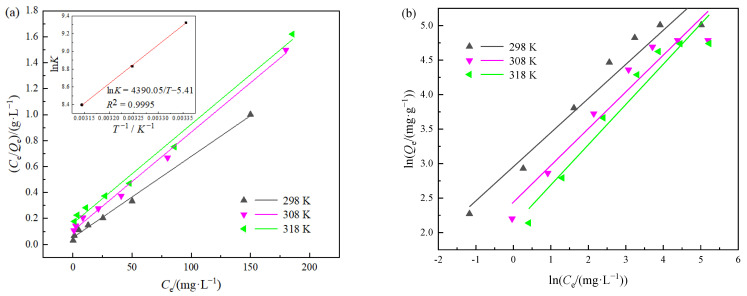
(**a**) Langmuir and (**b**) Freundlich adsorption isotherms for the adsorption of phenol on S2.

Fitting curves and adsorption isotherm constants for phenol adsorption on S2 are shown in Figure 8 and Table 3. According to the desired *R*^2^ (*R*^2^ > 0.99), the adsorption behavior of phenol on the adsorbent surface could be better described by the Langmuir adsorption isotherm equation. The thermodynamic parameters calculated for phenol adsorption on S2 are shown in Table 3. Δ*H* < 0, Δ*S* < 0, Δ*G* < 0, indicated that the adsorption was spontaneous adsorption, driven by enthalpy. As Δ*H*=−36.5 kJ·mol^−1^, which was a within the range of hydrogen bond adsorption enthalpy (2–40 kJ·mol^−1^) [55], it could be inferred that the adsorption of S2 to phenol might be dominated by the hydrogen bond adsorption of the carbonyl group to phenol. Because of the directivity and saturation of the hydrogen bond, the adsorption of phenol on the adsorbent surface was mainly monolayered. The *n* values fitted by the Freundlich adsorption isotherm equation were all above 1, indicating that the adsorption of phenol on S2 was a beneficial adsorption process. At 298 K, the *n* value was 2.0211, indicating that the adsorption of phenol on S2 could easily occur at room temperature.

### 3.4. Recovery Performance

**Figure 9:**
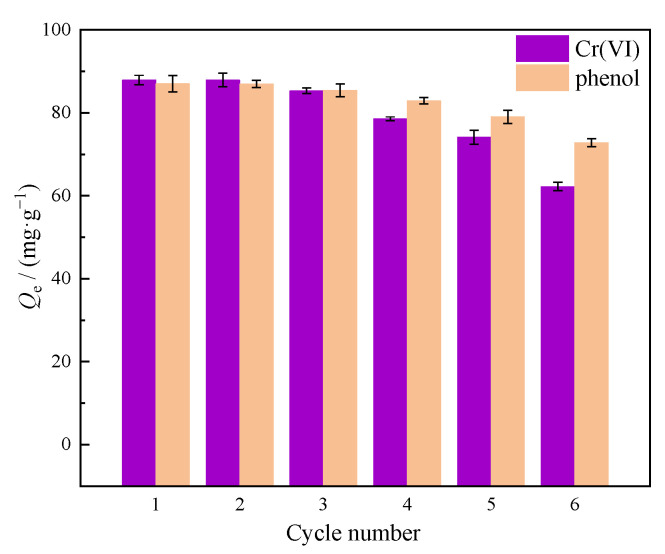
Adsorption–desorption cycle of Cr(VI) on S1 and phenol on S2.

Six adsorption–desorption cycles were carried out to evaluate the recyclability of the adsorbent, which is an important point for practical applications. Figure 9 represents the adsorption–desorption cycle of Cr(VI) on S1 and phenol on S2. For the first three cycles, the adsorption amount of Cr(VI) was 87.92 mg·g^−^^1^, 87.91 mg·g^−^^1^, and 85.32 mg·g^−^^1^, respectively, which showed stable adsorption performance of the hybrids to Cr(VI). After six adsorption-desorption cycles, the adsorption amount of Cr(VI) was 62.25 mg·g^−^^1^, which accounted for 70.80% of the initial adsorption amount. These results indicated that the adsorbent still possessed an adequate adsorption capacity toward Cr(VI) after six cycles. For phenol adsorption, the adsorption amounts were 87.02 mg·g^−^^1^, 86.98 mg·g^−^^1^, and 85.43 mg·g^−^^1^ for the first three cycles, respectively, which also showed stable adsorption performance of the hybrids toward phenol. After six adsorption–desorption cycles, the adsorption amount for phenol was 72.79 mg·g^−^^1^, which accounted for 83.65% of the initial adsorption amount. These results indicated that the adsorbent also possessed a good adsorption capacity toward phenol after six cycles.

### 3.5. Binary Systems Competitive Adsorption Behavior

**Figure 10:**
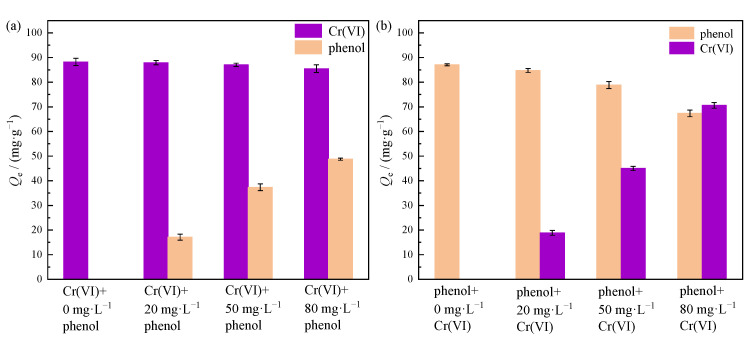
(**a**) The effect of the initial concentration of phenol on Cr(VI) adsorption and (**b**) the effect of the initial concentration of Cr(VI) on phenol adsorption.

**Figure 11:**
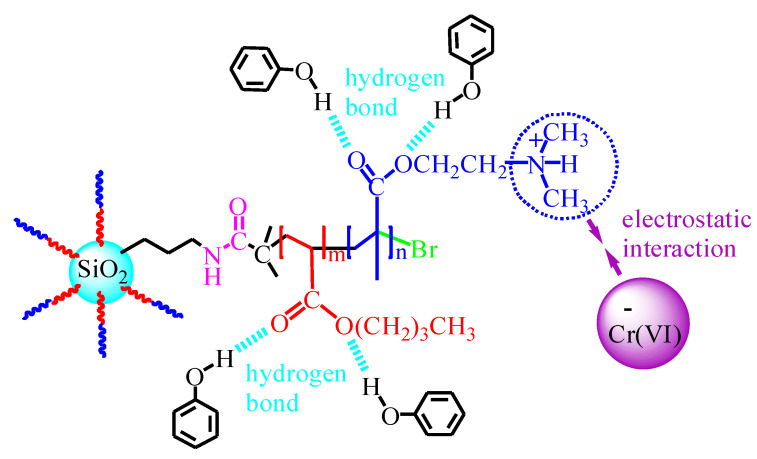
The proposed mechanisms for Cr(VI) and phenol adsorption.

The effect of the initial concentration of phenol on Cr(VI) adsorption is shown in Figure 10a. Phenol had little influence on the adsorption effect of Cr(VI). This was because when the pH < 7.8 [28,56], amino existed in the form of quaternary ammonium, and strong electrostatic attraction could have happened between the quaternary ammonium in SiO_2_-g-PBA-b-PDMAEMA and Cr(VI) (in the forms of HCrO_4_^−^ and CrO_4_^2^^−^). The presence of phenol almost did not affect the electrostatic adsorption of Cr(VI) on the quaternary ammonium groups. The effect of the initial concentration of Cr(VI) on phenol adsorption is shown in Figure 10b. With the increase of the initial concentration of Cr(VI), the adsorption capacity of phenol decreased gradually. This might be because potassium ion in potassium dichromate can form co-ordination bonds with carbonyl groups, which compete may have competed with phenol and reduced the adsorption active site for phenol. The presumed adsorption mechanism is shown in Figure 11. Strong electrostatic attraction between the positively charged quaternary ammonium in the PDMAEMA blocks and Cr(VI) existed as oxygen anion, as the main driving force for the Cr(VI) adsorption. Hydrogen bonds between ester groups in SiO_2_-g-PBA-b-PDMAEMA and phenol are the main driving forces for phenol adsorption. Generally, the silica-di-block polymer hybrids (SiO_2_-g-PBA-b-PDMAEMA) showed good adsorption performance toward Cr(VI) as well as phenol in both the single and binary systems. The adsorption capacity of the hybrids compared to other materials is given in Table 4 [5,6,7,14,57,58,59,60]. 

## 4. Conclusions

In this work, silica-di-block polymer hybrids, SiO_2_-g-PBA-b-PDMAEMA, with two ratios (SiO_2_/BA/DMAEMA = 1/50/250 and 1/60/240) were obtained via SI-ATRP methodology. All the results of the experimental studies can be summarized as follows:

(1) The results of FTIR and GPC proved that the SiO_2_-g-PBA-b-PDMAEMA hybrids were generated as expected;

(2) The adsorbent had excellent adsorption effects for Cr(VI) as well as for phenol. Furthermore, changing the proportion of hydrophilic and hydrophobic chain segments allowed for the adjustment of the adsorption performance of the adsorbents for water-soluble ions and organic pollutants. S1 (SiO_2_/BA/DMAEMA = 1/50/250) showed a higher removal efficiency of Cr(VI) (88.25%) than S2 (SiO_2_/BA/DMAEMA = 1/60/240, 79.88%), and S2 showed a higher removal efficiency of phenol (88.17%) than S1 (80.44%);

(3) Kinetics studies showed that the adsorption of Cr(VI) and phenol fitted the pseudo-second-order model well;

(4) The thermodynamic studies showed that the adsorption of Cr(VI) and phenol were all exothermic processes; therefore, decreasing the temperature was favorable for the adsorption of Cr(VI) and phenol on SiO_2_-g-PBA-b-PDMAEMA. The adsorption behavior of Cr(VI) and phenol was better described by the Langmuir adsorption isotherm equation, indicating that Cr(VI) and phenol were mainly adsorbed by monolayer. Thermodynamic parameters showed that the adsorptions were all spontaneous adsorption driven by enthalpy;

(5) The thermodynamic parameters suggested that the driving force of Cr(VI) adsorption on S1 was mainly the electrostatic attraction of anions by the quaternary ammonium of the PDMAEMA chains, while the adsorption of S2 to phenol was dominated by the hydrogen bond adsorption of carbonyl groups to phenol.

Further research will be conducted on simultaneous adsorption of various pollutants. 

## Figures and Tables

**Table 1:** Detailed recipes for prepared samples.

Sample (SiO_2_-Br:BA:DMAEMA)	SiO_2_-Br /mmol	BA /mmol	DMAEMA /mmol	CuCl /mmol	CuCl_2_ /mmol	PMDETA /mmol	CYC /g
S1 (1:50:250)	0.1620	8.1000	40.5000	0.1620	0.0162	0.1620	11.4773
S2 (1:60:240)	0.1620	9.7200	38.8800	0.1620	0.0162	0.1620	11.4066

Note: dosage of CYC was calculated based on a solid content of 40%.

**Table 2:** Fitting parameters of adsorption kinetics.

Systems	Adsorbents	*Q*_e,exp_ /mg·g^−1^	Pseudo-First-Order			Pseudo-Second-Order		
			*Q*_e,cal_ /mg·g^−1^	*k*_1_ /min^−1^	*R* ^2^	*Q*_e,cal_ /mg·g^−1^	*k*_2_ /g·mg^−1^·min^−1^	*R* ^2^
Cr(VI)	S1	88.25	18.88	0.04364	0.9055	90.66	0.00242	0.9988
	S2	79.88	37.98	0.04223	0.9688	83.19	0.00167	0.9971
phenol	S1	80.44	142.62	0.04313	0.9124	92.42	0.00038	0.9944
	S2	88.17	140.87	0.04821	0.9395	97.28	0.00054	0.9957

**Table 3:** Adsorption isotherm constants and thermodynamic parameters for Cr(VI) adsorption on S1 and phenol adsorption on S2.

Systems	Temperature /K	Langmuir			Freundlich			Thermodynamic Parameters		
		*Q*_m_ /mg·g^−1^	*K*_L_ /L·mg^−1^	*R* ^2^	*n*	*K*_f_	*R* ^2^	Δ*H* /(kJ·mol^−1^)	Δ*S* /(kJ·mol^−1^·K^−1^)	Δ*G* /(kJ·mol^−1^)
Cr(VI)	298	174.22	0.1114	0.9947	2.0617	21.3278	0.9541	−35.9	−0.0419	−23.4138
	308	140.06	0.0701	0.9970	1.8818	12.2125	0.9341			−22.9948
	318	139.08	0.0447	0.9940	1.7100	8.5612	0.9395			−22.5758
phenol	298	159.74	0.1191	0.9952	2.0211	19.1839	0.9385	−36.5	−0.0449	−23.1198
	308	131.58	0.0729	0.9949	1.8757	11.4701	0.9240			−22.6708
	318	130.89	0.0472	0.9906	1.7124	8.2102	0.9298			−22.2218

**Table 4:** Comparison of different adsorbent materials.

Systems	Adsorbent Material	*Q*_m_ of Cr(VI) (mg·g^−1^)	*Q*_m_ of Phenol (mg·g^−1^)	References
Cr(VI)	divinylbenzene copolymer resin	99.91		[5]
	coffee polyphenol-formaldehyde resin	175.44		[6]
	coffee polyphenol-acetaldehyde resin	143.32		[6]
	puresorbe	76.92		[7]
	silica-di-block polymer hybrids	174.22		This study
phenol	palm-tree fruit stones		129.56	[14]
	porous acrylic ester polymer		78.70	[57]
	polymeric adsorbents IRA-96C		59.85	[58]
	silica-di-block polymer hybrids		159.74	This study
Cr(VI) and phenol	iron incorporated rice husk	36.3817	6.569	[59]
	natural red clay modified by hexadecyltrimethylammonium bromide	4.47	1.13	[60]
	silica-di-block polymer hybrids	87.02	37.36	This study

Note: *Q*_m_ refer to saturated adsorption capacity.

## Data Availability

Data presented in this study are available on request from the first author.
